# Advances in the Pathogenesis of EBV-Associated Diffuse Large B Cell Lymphoma

**DOI:** 10.3390/cancers13112717

**Published:** 2021-05-31

**Authors:** Paola Chabay

**Affiliations:** Laboratory of Molecular Biology, Pathology Division, Multidisciplinary Institute for Investigation in Pediatric Pathologies (IMIPP-CONICET-GCBA), Ricardo Gutiérrez Children’s Hospital, Gallo 1330, Buenos Aires C1425EFD, Argentina; pchabay@conicet.gov.ar

**Keywords:** Epstein Barr virus, diffuse large B cell lymphoma, microenvironment, gene expression

## Abstract

**Simple Summary:**

The last World Health Organization classification of Tumours of Haematopoietic and Lymphoid Tissues defines a new category, EBV + DLBCL, NOS, which incidence varies among different populations. Given the oncogenic characteristics of both latent and lytic viral proteins, EBV could replace some of the cellular pathways and/or mutations observed in non EBV-associated cases. In addition, the virus may turn the tumor microenvironment in a tolerogenic, in order to promote tumorigenesis, and may have also influence on survival in specific populations. The analysis of EBV pathogenesis in DLBCL may exhibit new potential targets for DLBCL treatment.

**Abstract:**

Diffuse large B-cell lymphoma (DLBCL) is the most common non-Hodgkin’s lymphoma (NHL) in adults. Epstein–Barr virus (EBV) positive DLBCL of the elderly was defined by the World Health Organization (WHO) in 2008, it was restricted only to patients older than 50 years old, and it was attributed to immunesenescence associated with physiological aging. After the description of EBV-associated DLBCL in children and young adults, the WHO redefined the definition, leading to the substitution of the modifier “elderly” with “not otherwise specified” (EBV + DLBCL, NOS) in the updated classification, and it is no more considered provisional. The incidence of EBV + DLBCL, NOS varies around the world, in particular influenced by the percentage of EBV+ cells used as cut-off to define a case as EBV-associated. EBV has effect on the genetic composition of tumor cells, on survival, and at the recruitment of immune cells at the microenvironment. In this review, the role of EBV in the pathogenesis of DLBCL is discussed.

## 1. Introduction

Diffuse large B-cell lymphoma (DLBCL) is the most frequent non-Hodgkin’s lymphoma (NHL) in adults. The World Health Organization (WHO) in 2008 defined provisional entity of DLBCL, based numerous studies performed in Asian population, that was named ‘‘Epstein–Barr virus (EBV) positive DLBCL of the elderly”. These tumors occur in apparently immunocompetent patients usually older than 50 years old and have a worse prognosis than EBV tumors, and they were attributed to immunesenescence associated with physiological aging [1]. Afterward, several groups studied EBV-association with DLBCL in children and young adults, demonstrating that EBV positivity is also detected in those age groups, that exhibited a larger morphological spectrum, along with a better survival. In consequence, the term “elderly” was substituted in the 2017 classification, with “not otherwise specified” (EBV + DLBCL, NOS), and it is no more considered provisional [2,3]. EBV-positive DLBCL, NOS, is an EBV-positive clonal B-cell lymphoid proliferation. The NOS designation excludes other more specific types of EBV-positive lymphoma. Therefore, routine EBV testing is required for all DLBCL, NOS cases to define this specific entity [2]. In this review, EBV involvement in DLBCL pathogenesis will be discussed. 

## 2. EBV Association with Lymphoma

Cancers that are attributable to infections have a greater incidence than any individual type of cancer worldwide. The International Agency for Research on Cancer (IARC) classified 11 pathogens as carcinogenic agents in humans, which include EBV [4,5]. EBV is a gammaherpesvirus with striking biological properties. EBV establishes latent infection in lymphocytes, triggering the proliferation of the latently infected cells [6] once the virus was orally transmitted from the saliva of healthy carriers with latent persistent infection [7]. In the oropharyngeal epithelium, it enters the crypts, and passes through the epithelial cells layer to infect resting B cells, to drive them to become a proliferating lymphoblast. Then, as proposed by the “germinal center (GC)” model of viral infection, EBV-infected cells become involved in the GC reaction when they enter to the GC, where the virus downregulates the pattern of latent proteins towards to the default program [8,9]. Finally, the virus establishes a persistence in memory B cells for life, in which it could express alternatively the EBNA1 only program, or the latency program, with no viral proteins expressed at all [9]. Occasionally, due to the reactivation from latency into virus lytic cycle by several mechanisms, the virus replicates at the secondary foci at oropharyngeal sites, in order to ultimately disseminate to other hosts [10]. The successive downregulation of almost all EBV-latent proteins enables the infected cells to escape the immune recognition, thus becoming almost invisible to the immune system during viral persistence for lifetime. Furthermore, EBV is also able to replicate productively in epithelial cells, and besides, it has the ability to infect T cells and natural killer (NK) cells [7]. The infection on T cells is mediated by the viral glycoprotein gp350 and the CD21 receptor on T cells [11]. Furthermore, it was suggested that EBV type 2 exploits the T cell compartment to persist, and, in humanized mouse model infected by EBV2, both T cells and B cells are infected, and most animals develop a B cell lymphoma resembling DLBCL [12].

Both innate and adaptative immune responses are responsible for the viral immune control in the immunocompetent host during persistence and occasional reactivations, as a result of a delicate balance between infection and host immune response. NK cells are involved in the control of the first steps of primary infection, by the restriction of lytic infection, especially in children [13,14]. Both CD4 and CD8 T cell responses are implicated in the control of primary and the persistent cycles of infection. Primary EBV infection triggers in the blood large expansion of virus-specific CD8+ T cells, along with modest expansion of virus-specific CD4+ T cells. In persistent infection, in healthy carriers, CD8+ T cell population against lytic and latent EBV epitopes may constitute up to 2% and 0.5% of the CD8+ T cell population, respectively, while EBV-specific memory CD4+ T cells are stimulated to produce multiple cytokines following antigen challenge [15].

The low incidence of these EBV-associated lymphomas in immunocompetent individuals highlights the key role of the immune system mounted by carriers against EBV primary and persistent infection, in order to control and maintain the virus in the reservoir of persistent latent infection. However, since it induces the permanent proliferation of the infected lines in vitro, driven by the combined action of Epstein–Barr virus nuclear antigens (EBNAs) and latent membrane proteins (LMPs) [16], EBV could be associated with several lymphomas, especially if the balance between viral persistence and the immune response is disrupted. EBV is causally associated with B and T/NK lymphoproliferative diseases (LPD), and also with distinct tumors, namely: Burkitt lymphoma (BL), diffuse large B cell lymphoma (DLBCL), Hodgkin lymphoma (HL), plasmablastic lymphoma, (PBL), T/NK cell lymphomas, gastric carcinoma, nasopharyngeal carcinoma (NPC), leiomyosarcoma, and primary effusion lymphoma (PEL), in which both EBV and KSHV are present [17].

## 3. Epidemiology of EBV + DLBCL in Different Populations

EBV + DLBCL was initially described in Asian populations, restricted to patients older than 50 years old, reason why it was initially defined as a provisional entity, EBV + DLBCL of the elderly, by the WHO in 2008 [1]. Oyama et al. described EBV presence in 22 patients with large cell lymphoma without predisposing immunodeficiency, which expressed the EBV-encoded RNA (EBER) in the nuclei of the malignant cells [18]. Afterward, characterization of the EBV presence in DLBCL cases from Japan ranged from 3 to 14%; even though most studies included patients older than 50 years old, a few patients were younger [19,20,21,22,23,24]. When this study was extended to DLBCL in other Asiatic populations, the prevalence of EBV + DLBCL was 4–14% [25,26,27,28,29]. On the other hand, in Western countries, EBV association with DLBCL is restricted to less than 5% of cases [30,31,32,33]. Of note, in Latin America, Mexico, Peru, and Argentina, all developing populations where EBV infects young children mostly without symptoms [5], the prevalence of EBV + DLBCL in adults achieved 7% [32], 15% [34,35], and 9% [36], respectively. Furthermore, EBV was also associated to immunocompetent pediatric DLBCL patients from Argentina and Iraq [37,38] (Table 1).

**Figure 1:**
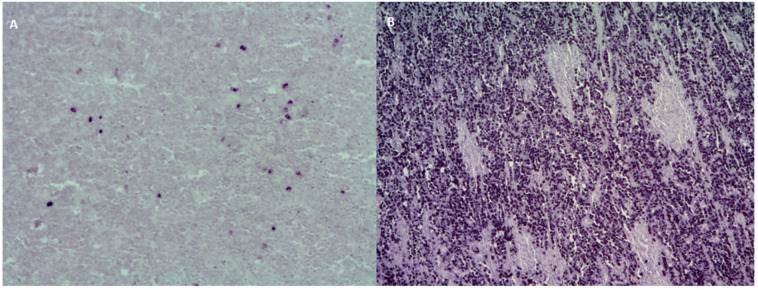
EBERs positive staining at the nucleus of (**A**) <10% of tumor cells and (**B**) 90% of tumor cells. Magnification 100×.

The differences in EBV prevalence in DLBCL among different populations might be associated to the epidemiology of EBV primary infection. In the context of underdeveloped populations, the first contact with EBV usually happens in the first decade of life and results in an asymptomatic infection, while in developed ones, it occurs mainly in adolescents or young adults, and it is symptomatic in about half of the cases, known as infectious mononucleosis (IM) [6]. Several factors are associated with the early acquisition of primary EBV infection, such as geographic region, race/ethnicity, and socioeconomic status [45]. However, this difference could be linked to the percentage of EBV+ cells used as a cut off (Table 1) (Figure 1). Even though the WHO revision in 2017 proposed that, with EBER in situ hybridization, more than 80% of the atypical cells may be positive to consider a DLBCL as EBV-associated [3], several studies suggested that EBV could be involved in lymphomagenesis process in cases below this cut off. For instance, Oyama et al. applied a cut off value of more than 50% EBERs+ cells [18], whereas Kuze et al. adopted the criteria of almost all tumor cells with positive signals as EBV + DLBCL [21]. In addition, Montes-Moreno et al. described a 10% cut off in their elderly DLBCL series [46], and Beltran et al. in young immunocompetent individuals from Perú [35]. Wada et al. extensively discussed the need for a uniform criterion for EBV positivity, either >20%, >50%, or almost all tumor cells, given that, in their series from Japan, when the cut off was >20% or >50%, the EBV-associated DLBCL cases were 3.3% or 1.0%, respectively, but they actually adopted the 20% of EBERs+ cells as cut off [23]. In line with this, Park et al. [20] and Hong et al. [29] referred >20% of tumor cells with positive signals by in EBERs ISH to define EBV + DLBCL in their series. In order to further explore EBV characteristics to define this cut off, Cohen et al. observed that cases above 20% of EBERs+ cells mostly displayed latency II and III patterns, while all the cases below expressed latency I antigens [36,37], suggesting that oncogenic latent viral proteins could also be involved in the pathogenesis of EBV + DLBCL. Moreover, a role in the pathogenesis was also attributed to EBV+ bystander cells, given that EBV + DLBCL patients and EBV − DLBCL patients with EBV+ bystander cells tended to have high and high-intermediate International Prognostic Index scores and poorer prognosis than EBV − DLBCL patients without EBV+ bystander cells [40]. In addition, traces of EBV infection were detectable by high-sensitivity methods in several EBV-associated lymphomas such as DLBCL, suggesting a “hit-and-run” mechanism [47]. Therefore, EBV may be involved in the pathogenesis of lymphoma more widely than recognized so far.

## 4. EBV Latent and Lytic Antigen Expression

EBV-associated lymphomas show a differential expression pattern of latent genes, which represent the pathological counterpart of EBV latent gene expression proposed by the “germinal center” model of infection. For instance, EBV-associated posttransplant lymphoproliferative disorders (PTLDs) usually express all the latent genes, known as the latency III program, that encode six nuclear (EBNA1, -2, -3A, -3B, -3C, and -LP) and three membrane (LMP1, -2A, and -2B) antigens, along with untranslated RNAs [48]. HL express a pattern, characterized by the expression of EBNA1 and LMP1 and LMP2 antigens, identified as latency II [49], while BL displays latency I pattern, that predominantly expresses EBNA1 [48] (Table 2). In all latency types, infected cells express two EBV-encoded small RNAs, known as EBER-1, and EBER-2 [48]. The latent viral antigens EBNA3A, EBNA3C, EBNA2, EBNALP, and LMP1 were established to be essential for efficient B-cell transformation [50], while the remaining latent antigens, as well as noncoding RNAs have an influence on B-cell transformation and maintenance of B-cell outgrowth [48]. Furthermore, several studies also demonstrated the importance of the lytic cycle, or at least its initiation, in supporting EBV-driven malignancies [51].

The growth-transforming capacity of EBV proteins, in particular the latent ones, drives the EBV-infected cells in proliferation by activating several pathways [17]. LMP1, the most important viral oncogenic protein, activates major cellular pathways to trigger B-cell transformation in vitro, including the ERK, JNK, and p38 signaling pathways, and the NF-kB pathway, whereas it also induces the expression of several cellular factors comprising CD21, CD40, ICAM1, LFA1, and other adhesion factors [16]. In addition, the remaining latent proteins also contribute to a greater or lesser extent to EBV-mediated tumorigenesis. LMP2A acts as a normal B cell receptor (BCR), providing a tonic survival signaling to B cells, which, even in the absence of a BCR, are able to drive GC formation [8,52]. When coexpressed with LMP1 at the GC, they modulate each other. In fact, LMP2A alone would ensure that the cells form GCs, then LMP1 and LMP2A together provide the requisite survival signals, to end with the LMP1 expression alone, that ensures exit from the GC and terminal differentiation [8]. On the other hand, EBNA2 induces the transcription of the cellular oncogene MYC, which induce a proliferative state along with apoptosis. However, the cooperation of EBNA3A and EBNA3C rescue infected cells from apoptosis via the downregulation of the proapoptotic BIM and p16INK4a proteins [51]. Viral EBNAs also target a wide range of cellular processes from genome maintenance, gene expression, cell cycle regulation, and tumor suppression, to contribute to the EBV mediated B-lymphocyte transformation. EBNA1 specifically ensures the segregation of viral genome to daughter cells during mitosis of EBV-infected cells [16]. The non-coding RNAs expressed by EBV include, besides the two EBERs, 44 miRNAs, which has an impact on several signaling pathways essential for cell survival to optimize EBV-mediated B cell transformation [51].

Given the fact that EBV + DLBCL was initially described in elderly patients, and immunosenescence was proposed as a pathogenic factor for deregulation of EBV-transforming proteins, the expression of all viral proteins, the latency III pattern, was presumed. However, in the first provisional entity in 2008 and in the revised version in 2017, both latency II and latency III profile were observed. In fact, EBNA2 and EBNA3A expression, which defines latency III pattern, was demonstrated in elderly cases [18,19,21,31,53], and in the context of immunosuppression [33]. Nevertheless, both latency II and III patterns were described in young immunocompetent DLBCL patients [36,54], and in children with EBV + DLBCL, NOS [37,38]. Even though latent viral antigens were associated with several degrees of transforming capacity, lytic cycle, or at least its first steps, could be involved in lymphomagenesis. In fact, it was proposed that lytic replication in EBV-associated malignancies is not directly linked to EBV particle production, but also has influence on the tumor microenvironment that promotes tumorigenesis [53]. In EBV + DLBCL, immediate-early BZLF1, early BHRF1 and BMRF1, late BLLF1 lytic viral genes expression was detected, and their expression was correlated with IL10 and IFNγ expression, revealing a link between viral lytic cycle and EBV + DLBCL pathogenesis [55].

## 5. Cellular Gene Expression

Two subtypes of EBV + DLBCL, NOS were identified. The most frequent polymorphous subtype, in which medium neoplastic cells with Hodgkin/Reed–Sternberg-like cells are distributed in a reactive background with histiocytes, lymphocytes, and plasma cells. In contrast, the monomorphic subtype is characterized by large neoplastic cells with centroblastic or immunoblastic morphology, without a polymorphous inflammatory background. Large areas of geographical necrosis or apoptosis can be observed in both subtypes [56,57]. In EBV + DLBCL, NOS the neoplastic cells are usually positive for the B-cell antigens CD19, CD20, CD22, CD79a, and PAX5 [3]. In addition, CD30 is frequently positive and CD15 is coexpressed in a few cases [3]. In EBV + DLBCL, NOS, the activation of the JAK/STAT and NF-kB pathways, which triggers the expression of phosphorylated STAT3 and NF-kB, are more frequent in comparison with EBV-negative DLBCL [41,56]. Approximately 60% of cases display clonal rearrangement of the immunoglobulin gene [41], and clonality assessment has been proved to be helpful for discriminating polymorphous cases from reactive hyperplasia [2,3].

Most B cell non-Hodgkin lymphomas (B-NHLs) are derived from GC B cells, including follicular lymphoma, BL and DLBCL, which together account for 80% of B-NHLs, as revealed by the presence of somatically mutated immunoglobulin genes in their genomes. DLBCL represents arrested B cells induced by different transformation events that occur at various stages of the GC transit. The cell-of-origin (COO) classification revealed that the germinal center (GC)-like subtype of DLBCL look like light zone B cells, whereas activated B cell (ABC)-like DLBCL originate from GC cells arrested during the early stages of post-GC plasma cell differentiation [58]. Furthermore, GC DLBCL was shown to be related with normal GC B cells that are in the light zone early or intermediate stages, while most ABC-DLBCL may originate from cells that are not yet committed to plasmablastic differentiation [59]. The COO classification is associated with distinct clinical outcomes, with GCB cases being, usually, less aggressive than ABC cases [60]. The COO classification in DLBCL was assessed by two different approaches. In one hand, GC and ABC subtypes were defined by gene expression analysis (GEP), even in formalin fixed paraffin embedded samples, using different technologies such as Lymph2X, which identified patient groups with significantly different outcomes after R-CHOP [61], particularly in the presence of BCL2 alterations [62]. When GEP analysis is not available in clinical practice, the immunohistochemistry (IHC) based Hans algorithm [63] can be used as a surrogate marker, since it displays an overall concordance of 72% with GEP [64]. In EBV + DLBCL characterized with Hans immunohistochemical markers, ABC-associated proteins IRF4, MUM1, are typically positive, whereas GC markers CD10 and BCL6 are usually negative. In line with this, GEP analysis confirmed ABC prevalence in EBV-associated cases [41,65,66], even in children [38]. However, in several populations, no significant difference was observed in EBV + DLBCL between the GCB and ABC subtypes [67,68]. The predominance of ABC may be originated by the LMP1 latent viral protein, expressed in both latency II and III patterns, which mimics a CD40 receptor constitutively activated, that in turn induces the activation of the NF-kB /IRF4 pathway [46], thus leading to BCL6 downregulation [69]. Furthermore, gene expression profile (GEP) in EBV + DLBCL revealed that the JAK-STAT and NF-kB pathways were enriched in EBV + DLBCL, and this finding was confirmed in vitro in cell lines, and also in patients by immunohistochemical staining [39]. In addition, GEP by microarray analysis proved 148 differentially expressed genes, including 97 upregulated and 51 downregulated genes in EBV + DLBCL compared with EBV − DLBCL [70].

## 6. Genetic Alterations

Advances in molecular biology led to understand the key oncogenic pathways involved in the biological diversity of DLBCL. Next-generation sequencing (NGS) technologies identified unique molecular targets that may be exploited for therapy [71]. Genetic alterations in DLBCL have been extensively reviewed, including the differences in GC and ABC subtypes ([71,72,73] and references herein). The EBV-related transforming mechanisms may replace, or at least complement, the genetic alterations involved in DLBCL development, and studies comparing EBV+ and EBV− cases were performed to enlighten the role of EBV.

Contribution of EBV to DLBCL pathogenesis was proven to be marked regarding chromatin remodeling MYD88 and/or CD79B genes mutations, given that their presence is almost mutually exclusive with EBV infection, suggesting that they could represent distinct DLBCL subgroups with different oncogenic drivers [74]. In addition, CARD11 and EZH2 missense mutations were described in a few elderly patients with EBV + DLBCL [75]. In line with this, in a large study of EBV-related lymphoma, specifically EBV + DLBCL cases were characterized by frequent TET2 and DNMT3A mutations and the paucity of MYD88, CD79B, CDKN2A, and FAS alterations [76]. Zhou et al. analyzed 9 EBV+ and 6 EBV − DLBCL cases by NGS, and they reported MYC, RHOA, PIM1, MEF2B, MYD88, and CD79B mutations in about 25% of EBV + DLBCL, compared with KMT2D, CREBBP, PIM1, TNFAIP3, and BCL2 mutations observed in 35–65% of EBV − DLBCL cases [77]. In a series of 11 EBV + DLBCL cases, Liu et al., described mutations in exonic regions ranging from 3.49% ± 22.68%, identifying 57 selected distinct candidate variants, and confirmed that the 10 most frequent mutations were in PRSS3, MUC3A, MUC16, MUC19, RLIM, HERC2, BCAR3, PRSS1, RPA1, and AMD1 [78].

Concerning rearrangements and copy number alterations (CNA), fewer MYC, BCL2, and BCL6 rearrangements were observed in EBV + DLBCL [74,75], reinforcing the role of EBV as the alternative pathway to trigger DLBCL pathogenesis. Furthermore, fewer genomic alterations were described in a series of EBV + DLBCL compared with those observed in EBV − DLBCL cases, being the most frequent CNAs (copy number alterations) (>30%) in EBV + DLBCLs losses at 6q27, 7q11.2, and 7q36.2–36.3; and gains at 1q23.2–23.3, 1q23.3, 1q32.1, 5p15.3, 8q22.3, 8q24.1–24.2, and 9p24.1 [71]. Additionally, genome-wide high-resolution screening for CNAs in a series of 24 EBV + DLBCL samples revealed copy number gains, 1p36.33, 2p11.2, 6p22.1, 6p22.2, 6q14.3, 9p13.3, 10p11.22, 14q32.33, 17p13.1, 20p11.22, and 20p13, whereas cytogenetic losses involved regions 1p36.33, 2p11.2, 2q37.3, 8q24.3, 10q26.3, 15q11.1–q11.2, 16p11.2, 20q13.33, and 21q11.2 [75]. In contrast, the EBV presence could be associated with the upregulation of specific genes involved in immune evasion, such as PDL1 and PDL2. In fact, EBV presence in tumor cells was proposed as an alternative mechanism for PDL1 induction, given the fact that 9p24.1 amplification, the gene that encodes for PDL1, and EBV infection were proved to be mutually exclusive in a series of Hodgkin lymphoma [79]. In line with this, high frequency of PDL1/PDL2-involving genetic aberrations was observed in EBV-associated lymphomas, including extranodal NK/T-cell lymphoma, aggressive NK-cell leukemia, systemic EBV-positive T-cell lymphoproliferative disorder, peripheral T-cell lymphoma, not otherwise specified, and EBV + DLBCL [76,80]. Moreover, it was suggested that the upregulation of PDL2 on 9p24.1 may induce immune evasion, and it was associated with poor prognosis in EBV + DLBCL [70]. The amplification of PDL1 could be involved in the increased expression of PDL1, detected by immunohistochemistry (IHC), in tumor cells in EBV + DLBCL, which was also proved to be associated with prognosis [54,81,82,83]. In fact, it was demonstrated that EBV − LMP1 increases PDL1 promoter and enhancer activity [84].

## 7. Microenvironment Composition

EBV can induce a highly variable composition of the tumor microenvironment (TME), depending on the EBV-associated tumors. The most widely studied is EBV + Hodgkin lymphoma (HL), where TME plays a key role. Even though EBV + tumor cells are not as immunogenic as lytically infected ones, they are capable of eliciting EBV− specific immune responses. In fact, it has been demonstrated that EBV presence elicits a higher number of infiltrating CD4+ T cells, in particular Treg cells that secrete IL10, and activated cytotoxic CD8+ T cells and NK cells [85]. Besides, in HL, significantly higher numbers of tumor associated macrophages were observed in EBV+ HL as compared to EBV-cases [85,86]. Furthermore, immune scape in EBV-associated cancers can be triggered by overexpression of PDL1/PDL2 and CTLA-4, which induce T cell exhaustion [79,87,88,89].

Regarding EBV-associated DLBCL, strong PDL1 expression in tumor cells of patients with EBV+DLBCL was observed, suggesting a mechanism of induction of immune tolerance [83]. Nevertheless, given that PDL1 can be also expressed in immune cells at the microenvironment, PDL1 expression was also demonstrated in the TME of EBV + DLBCL cases, as a mechanism to strengthen immune exhaustion. Kiyasu et al. discriminated PDL1 expression in tumor cells and at the microenvironment, and described that PDL1 + cells at the TME was significantly associated with EBV positivity, whereas, unexpectedly, the number of the PD1+ tumor infiltrating lymphocytes, the ligand of PDL1, was not related to EBV presence [90]. In line with this, in young patients EBV + large cell lymphomas displayed dysregulation of immune checkpoints, IDO, and PD1/PDL1 axis, promoting a tolerogenic immune environment [54]. PDL1 expression in nonmalignant cells comprised mainly tumor-infiltrating macrophages, in EBV + DLBCL, and in other lymphomas, by double immunostaining for CD68 or PAX5 and PDL1 [91]. PDL1 expression in macrophages could be induced as a response to IFNγ, an antitumoral and antiviral cytokine [84]. Furthermore, in patients with EBV+ posttransplant lymphoproliferative disorders (PTLD), latency II or III profiles were strongly associated with PDL1 expression in tumor associated macrophages (TAMs) compared with EBV− cases, and also with cases expressing latency I [92], suggesting that latency II and III viral genes, such as LMP1, could be involved in PDL1 upregulation. Increased PDL1 expression was also observed in EBV + DLBCL cell lines, which increased the expression of PD1 on T cells in vitro, with the subsequent decreased on T cell proliferation. The intratumoral PD1+ T cells were mostly exhausted CD8+ memory T cells that produced more IL-2, IFN-γ, IL-10, and TNF-α after PD1 blockade [93].

The immune regulatory environment observed in EBV + DLBCL also involves increased gene expression of immunosuppressive cytokine IL10 [68], which could be triggered directly by LMP1 [93], and/or lytic antigens [55], or, alternatively, by the presence of regulatory T cells that secrete IL10, as observed in EBV-associated HL, nasopharyngeal and gastric carcinomas, and in EBV-transformed B cell lines [84,94]. However, in EBV + DLBCL the regulatory environment coexists with the increased expression of CD8+ T-cells and granzyme B+ cytotoxic effector cells [68], also known as “inflamed phenotype” by gene expression analysis [72]. Nevertheless, the CD8+ T cell in the TME might not be efficient to eliminate EBV-infected cells, since decreased numbers of central and effector memory CD8+ T lymphocytes were found among EBV + DLBCL patients [95]. The presence of immune tolerogenic environment in the context of EBV + DLBCL was also described by gene expression analysis, which revealed increased expression of PDL1, PDL2, LAG3, and TIM3 immune checkpoints and a higher protumoral CD163/CD68 “M2” macrophage polarization pattern [96]. Therefore, even though EBV-associated tumors are frequently characterized by an important inflammatory infiltrate, it may represent an ineffective attempt of the host immune response to control virus-infected tumor cells [97].

The expression of EBV latent antigens, in particular latency II and III observed in EBV + DLBCL, modulates the environmental immune response by a several mechanisms. In EBV-transformed B lymphoblastoid cell lines that represents DLBCL, viral latency increased the production of IL10, CCL22, and MIP-1α/CCL3 [98]. LMP1 also promotes B-cell proliferation by inducing a STAT1-dependent IFN-γ secretion [99], whereas both LMP2A and EBERs latent antigens, and BZLF1 lytic one, enhance IL10 production [97,100,101,102], highlighting the relevance of IL10 expression in B-cell transformation. Upregulation of IL6 and IL13, triggered by viral antigens such as EBER2 and BZLF1, may also induce the formation of niches favorable for the growth and survival of EBV-infected tumor cells [101,103].

## 8. Survival

The response to combination chemotherapy seems to be lower in EBV + DLBCL than in EBV-negative DLBCL cases. The treatment with the anti-CD20 (rituximab) in addition to anthracycline-based chemotherapy has clearly improved survival outcomes in patients with DLBCL in several clinical settings. In fact, the overall response with R-CHOP varies from 50 to 89% in different series [104].

The prognosis in EBV + DLBCL differs between elderly and young patients [3]. Overall survival was thus significantly lower in elderly EBV + DLBCLs [22,28,29,34,46,105,106], and an age older than 70 years was independently predictive for survival in multivariate analysis [19]. In patients younger than 50 years, EBV positivity did not markedly influence overall or progression free survival, but there was a trend toward poorer survival in EBV- associated cases older than 50 years. In both univariate and multivariate analysis, EBV presence displayed an adverse impact in ABC patients with DLBCL, but it did not influence survival in GCB cases [20,46]. Furthermore, markedly worse survival rate was noted for DLBCL cases displaying viral latency III pattern [31], and in EBV + DLBCL patients and EBV − DLBCL patients with EBV+ bystander cells, compared to patients without any detectable EBV+ cells [40]. Nicolae et al. also demonstrated, in patients with large B cell lymphoma, that younger ones achieved a significantly higher overall survival than prior series of EBV+ elderly cases [54]. Conversely, in elderly and young groups, EBV + DLBCL patients showed significantly worse survival than negative cases [27,69], and also no significant differences in clinical outcomes were identified between different age groups with EBV positive DLBCL [27,42,107].

Quite the opposite, other studies described no significant impact of EBV on survival in DLBCL patients [23,33,41,43,44,75,108]. Nevertheless, a few studies enlighten the effect of EBV on prognosis in relation to other markers. Ok et al. revealed that EBV + DLBCL with that also express CD30 had significantly lower overall survival compared with EBV + DLBCL without CD30 expression [41]. In addition, PDL1+ expression in a subset of EBV + DLBCL displayed inferior clinical outcome compared with PDL1− cases, suggesting a mechanism for immune evasion through PDL1 to underlie the worst outcome of EBV + DLBCL [81,82]. Furthermore, upregulation of PDL2 on 9p24.1 also triggers immune evasion, and was proved to be associated with poor prognosis in EBV + DLBCL [70].

## 9. DLBCL Associated with Chronic Inflammation

This DLBCL is a lymphoid neoplasm frequently associated with EBV infection, initially described in Japan, and arises in the setting of longstanding chronic inflammatory process such as chronic osteomyelitis, pyothorax, metallic implant, or chronic skin ulcers [2,44]. Furthermore, it was also described in ileal neobladder, and related to breast implants [109,110,111]. The neoplastic cells have immunoblastic, centroblastic, or less commonly anaplastic features, with few nucleoli and limited to moderate amounts of cytoplasm. The proliferation rate achieves 80–100% [112]. Most lymphoma cells express CD20 and CD79a, but a proportion of cases could show plasmacytic differentiation, with a loss of CD70a and/or CD20, accompanied by the expression IRF4/MUM1 and CD138 markers. The lymphoma cells exhibit an activated B-cell phenotype [2]. DLBCL associated with chronic inflammation usually have a complex karyotype, with abundant numerical and structural abnormalities. MYC amplification is quite frequent and TP53 mutations are observed in approximately 70% of cases. The immunoglobulin genes show clonal rearrangements and evidence of somatic hypermutation [2,112]. NGS in a series of chronic inflammation DLBCL associated with breast implant described mutations in genes known to be recurrently mutated in DLBCL, such as CREBBP, GNA13, TET2, IRF4, STAT3, and SOCS1 [110]. Latency III program is prevalent, since viral oncogenic antigens EBERs, LMP1, and EBNA-2 are positive in most cases, which may be related to the pathogenesis of this particular DLBCL subtype [44,111]. Chronic inflammation at the local site perhaps plays a role in the proliferation of EBV-transformed B cells, since it allows them to escape from the host immune surveillance, as a result of the production of immunosuppressive IL10 cytokine, and the autocrine to paracrine growth via IL6 and IL6R [2]. Furthermore, EBV + DLBCL associated with chronic inflammation expresses CCL17 and CCL22 chemokines that are involved in the recruitment of CCR4-expressing regulatory T cells at the microenvironment [113]. In addition, downregulation of HLA class I expression, and mutations of cytotoxic T-lymphocyte epitopes in EBNA3B, an immunodominant antigen for cytotoxic T-lymphocyte responses, could also contribute to escape of neoplastic cells from host cytotoxic T lymphocytes [2].

Included in this category is the subtype fibrin-associated diffuse large B cell lymphoma, which is different from DLBCL associated with chronic inflammation category, given the fact that it does not form a discrete mass, and the clinical outcome is highly favorable [114].

## 10. Conclusions

In summary, EBV + DLBCL, NOS, is an aggressive lymphoma subtype associated defined by the recent WHO classification with a different impact on prognosis, according to population background. Its definition prompt the standardization of EBERs in situ hybridization as a routine test in pathological samples with suspected DLBCL diagnosis. Even though the cutoff for EBER positivity considers that more than 80% of the atypical cells may be positive to consider a DLBCL as EBV-associated [3], several groups discuss this definition, based on latent or lytic antigen expression [23,35,36,37,55], or the presence of EBV+ bystander cells in EBV − DLBCL [40]. Specific signaling pathways, such as JAK-STAT and NF-kB pathways are enriched in EBV + DLBCL. In contrast, MYD88 and/or CD79B mutations, and MYC, BCL2, and BCL6 rearrangements are absent in EBV+ cases [3]. To add more, EBV + DLBCL also promotes an exhausted immune microenvironment, by the dysregulation of immune checkpoint PD1/PDL1, LAG3, and TIM3 [54,97], the shift to a higher pro-tumoral CD163/CD68 “M2” macrophage polarization pattern [97] or the increase of immunosuppressive cytokine IL10 [68]. Therefore, studies are required to explore EBV antigens expressed in DLBCL, cellular pathways activated by viral presence, or microenvironment composition, in order to identify potential targets to treat this specific entity.

## Figures and Tables

**Table 1:** EBV + DLBCL in different populations.

Study	EBV + DLBCL Incidence	EBV + Cells Cut Off	Patients’ Origin	Latency Type	EBV Effect on Survival
Oyama et al., 2007 [18]	5.5%	>50%	Japan	II/III	60% (17 months) ^#^
Oyama et al., 2007 [19]	14%	>50%	Japan	II/III	EBV + inferior survival
Park et al., 2007 [20]	9%	>20%	Korea	NA	EBV + inferior survival
Sato et al., 2014 [22]	6.9%	>30%	Japan	II/III	EBV + inferior survival
Wada et al., 2011 [23]	5.2%	>20%	Japan	NA	No difference
Yamauchi et al., 2007 [24]	2%	NS	Japan	NA	NS
Pan et al., 2013 [25]	3.8%	>50%	China	NA	EBV + inferior survival
Lu et al., 2015 [27]	14%	>20%	China	NA	EBV + inferior survival
Chang et al., 2014 [28]	4.5%	>10%	Taiwan	II/III	EBV + inferior survival (trend)
Hong et al., 2015 [29]	8.4%	>20%	Korea	NA	EBV + inferior survival
Gibson et al., 2009 [30]	5.3%	80%	USA	NA	NS
Hoeller et al., 2010 [31]	3.1%	>10%	Switzerland, Italy, Austria	II/III	EBV + inferior survival
Hofscheier et al., 2011 [32]	8.1%	90%	Mexico	II/III	NS
Tracy et al., 2018 [33]	4.4%	>30%	USA	NA	No difference
Beltran et al., 2011 [34]	19.6%	>20%	Peru		40% (3 years) ^#^
Cohen et al., 2014 [36] and 2017 [39]	9.3%	>20%	Argentina	II/III	EBV + inferior survival
Uccini et al., 2015 [38]	54%	NS	Irak	II	2/6 CR ^#^
Ohashi et al., 2017 [40]	4.5%	>80%	Japan	NA	EBV + inferior survival
Ok et al., 2014 [41]	4%	>10%	USA	NA	No difference
Lu et al., 2014 [42]	16.9%	>20%	Taiwan	NA	No difference
Ahn et al., 2013 [43]	8.1%	>50%	Korea	NA	EBV + inferior survival
Beltran et al., 2018 [44]	28%	>20%	Peru	NA	54% (5 years) ^#^

Abbreviations: NS, not specified. NA, not assessed. ^#^ Survival evaluated only in EBV + cases.

**Table 2:** EBV latency patterns in viral associated malignancies.

EBV-Associated Neoplasia	Latency Type	Viral Antigen Expression
Burkitt lymphoma (sporadic and endemic)	I	EBERs,miRNAs, EBNA1
Gastric carcinoma	I	EBERs,miRNAs, EBNA1
NK/T cell lymphoma	I	EBERs,miRNAs, EBNA1
Plasmablastic lymphoma	I	EBERs,miRNAs, EBNA1
Primary effusion lymphoma	I	EBERs,miRNAs, EBNA1
Hodgkin lymphoma	II	EBERs,miRNAs, EBNA1, LMP1, 2A, 2B
Nasopharyngeal carcinoma	II	EBERs,miRNAs, EBNA1, LMP1, 2A, 2B
Diffuse large B cell lymphoma	II	EBERs,miRNAs, EBNA1, LMP1, 2A, 2B
Diffuse large B cell lymphoma	III	EBERs,miRNAs, EBNA1, LMP1, 2A, 2B, EBNA2, EBNA3A, 3B, 3C, LP
Postransplant lymphoproliferative disorder	III	EBERs,miRNAs, EBNA1, LMP1, 2A, 2B, EBNA2, EBNA3A, 3B, 3C, LP

Abbreviations: EBERs, Epstein Barr encoded RNAs; miRNA, microRNAs; EBNA1, Epstein Barr Nuclear Antigen 1; EBNA2, Epstein Barr Nuclear Antigen 2; EBNA3, Epstein Barr Nuclear Antigen 3; LMP1, latent membrane protein 1; LMP2, latent membrane protein 2.
